# Experimental indications of gardeners’ anecdotes that snails interfere with invasive slugs

**DOI:** 10.7717/peerj.11309

**Published:** 2021-05-11

**Authors:** Daniel Dörler, Verena Dorn, Theresia Widhalm, Micha Horacek, Florian Heigl, Pia Euteneuer, Friedrich Leisch, Thomas Frank, Johann G. Zaller

**Affiliations:** 1 Institute of Zoology, University of Natural Resources and Life Sciences, Vienna, Vienna, Austria; 2 Höhere Bundeslehr- und Forschungsanstalt Francisco Josephinum, BLT Wieselburg, Wieselburg, Austria; 3 Experimental Farm Gross-Enzersdorf, Department of Crop Sciences, University of Natural Resources and Life Sciences, Vienna, Gross-Enzersdorf, Austria; 4 Institute of Statistics, University of Natural Resources and Life Sciences, Vienna, Vienna, Austria

**Keywords:** Invasive species, Invasion biology, Aboveground-belowground-interactions, Snail-slug-interaction, Stable isotope tracing

## Abstract

The invasive Spanish slug (*Arion vulgaris*) is an important pest species in agriculture and horticulture in Europe. In the last decades it has spread across the continent where it outcompetes native slug and snail species, thus posing a threat for biodiversity. A popular anecdote suggests to promote Roman snails (*Helix pomatia*) in gardens because they are able to control *A. vulgaris*. We examined a potential interrelationship between these two species using a mesocosm experiment with lettuce plants. ^13^C-^15^N stable isotope labelling of lettuce allowed us to investigate interactions between *Helix* and *Arion* on weight gain/loss and herbivory. Additionally, we wanted to know whether different watering regimes (daily vs. every 3rd day watering of weekly amount) and earthworms alter these interactions. Egg predation of *Helix* on *Arion* eggs was further tested in a food-choice experiment. *Arion* showed a five times higher herbivory per body mass than *Helix* in a single-species setting. However, in mesocosms containing both species percentage of herbivory per body mass was significantly lower than in *Arion*-only mesocosms, especially when watered every three days. Overall isotope uptake via eaten lettuce was unaffected by the presence of the other species. Only very little predation (three out of 200 eggs) of *Helix* on *Arion* eggs was observed. Our results provide no evidence for a clear dismissal or confirmation of the popular gardener’s anecdote that *Helix* snails have a negative effect on *Arion* abundance or herbivory.

## Introduction

Invasive alien species are a growing concern for biodiversity, ecosystem services, economy and health worldwide (e.g. Doherty et al., 2016; Paini et al., 2016; Walsh, Carpenter & Van der Zanden, 2016; Balvanera et al., 2019; Kumar Rai & Singh, 2020). They can outcompete native species, transmit diseases, and negatively influence ecosystem services including agronomy (Simberloff, 2013). In Europe the only mollusc among the 100 worst invasive alien species is the Spanish slug *Arion vulgaris*, formerly known as *A. lusitanicus* (Rabitsch, 2006). The exact area of origin for *A. vulgaris* is still not definitely determined (Pfenninger et al., 2014; Zemanova, Knop & Heckel, 2014) and a recent study suspects it to be in France and Western Germany (Zając et al., 2020). Over the last decades, however, the slug has spread over large parts of Europe (Engelke et al., 2011; Kozłowski & Kozłowski, 2011; Hatteland et al., 2013; Papureanu, Reise & Varga, 2014; Balashov et al., 2018). In Austria, *A. vulgaris* was first described in 1972 from one lowland region only (Reischütz & Stojaspal, 1972) but nowadays seems to occur in gardens all over the country (Dörler et al., 2018).

*Arion vulgaris* feeds on many different plant species (Briner & Frank, 1998; Kozłowski & Kałuski, 2004; Kozłowski, 2005; Kozłowski & Jaskulska, 2014) and outcompetes native slug species (Kappes, 2006; Rabitsch, 2006; Slotsbo et al., 2012) or replaces them through introgression (Roth, Hatteland & Solhøy, 2012; Allgaier, 2015; Hatteland et al., 2015; Zemanova, Knop & Heckel, 2017). Potential reasons for this invasiveness lie in its endurance of adverse climate conditions (Slotsbo et al., 2011, 2012, 2013; Slotsbo, Hansen & Holmstrup, 2011) and higher reproduction rates than native species under these conditions (Knop & Reusser, 2012).

The Spanish slug can impact biodiversity, but is also a significant pest species in agriculture and horticulture, numerous chemical and biological control methods have been established (Barnes & Weil, 1942; Wilson et al., 1995; Friedli & Frank, 1998; Speiser, Zaller & Neudecker, 2001; Iglesias & Speiser, 2001; Speiser & Kistler, 2002; Grimm, 2002; Henderson & Triebskorn, 2002; Iglesias, Castillejo & Castro, 2003; Rae et al., 2007; Rae, Robertson & Wilson, 2009; Hass et al., 2010; Grubišić et al., 2018; Dörler, Scheucher & Zaller, 2019). Beyond these control methods, also biotic factors such as plant diversity (Trouvé et al., 2013; Zaller et al., 2013) or mycorrhizal fungi (Trouvé et al., 2013) can impact their herbivory activity. Even earthworms can significantly decrease slug herbivory by supporting plant defense mechanisms (Trouvé et al., 2013; Zaller et al., 2013), can alter the effectiveness of slug control methods (Gavin et al., 2012) and can serve as phoretic hosts for parasitic nematodes of slugs (MacMillan et al., 2009).

Since decades anecdotes circulate among gardeners postulating that the Roman snail *Helix pomatia* can control the invasive *A. vulgaris*, either by direct competition or by egg predation. Carnivory is known from several gastropod species (Barker & Efford, 2009) including the slugs *Limax maximus* (Lehmann, 1873), *Plutonia atlantica* (Wiktor & Backeljau, 1995) or several snail species of the *Zonitidae* are known to be at least facultative carnivores (Taylor, 1914). *Oxychilus cellarius* has also been described to feed on *Arion ater* eggs (Taylor, 1914). The Roman snail is widely spread in Europe (Meisenheimer, 1912; Järvinen et al., 1976; Neubert, 2013, 2014; Egorov, 2015) and can be found in woodlands, dry meadows, gardens and vineyards (Meisenheimer, 1912; Pollard, 1975; Neubert, 2013). In general, snails can cope better with drier periods than slugs due to their shells (Thompson et al., 2006). Former studies have shown that freshly hatched *H. pomatia* eat their empty shell cavities and sometimes even feed on the eggs of their siblings, leading to egg-cannibalism (Baur, 1988, 1990a). Individuals that feed on eggs have been shown to grow faster and were more likely to reach maturity than individuals that do not feed on eggs (Baur, 1990b). A characteristic for species that conduct egg-cannibalism is hatching asynchronicity (Desbuquois, Chevalier & Madec, 2000).

Hence, we investigated potential influences of *H. pomatia* on *A. vulgaris* and wanted to know to what extent this interaction is altered by abiotic (soil humidity) and biotic (earthworms) factors. We designed a factorial greenhouse experiment with lettuce as model plant, tracked species-specific herbivory using stable isotopes and assessed egg predation in a separate food-choice-experiment. To the best of our knowledge this is the first study that systematically investigates this gardeners’ anecdote in combination with other factors.

## Materials & methods

We set up two experiments in order to examine various interactions between *Helix* and *Arion*: first, a greenhouse experiment to examine a potential mutual influence between *Helix* and *Arion* individuals, and second, a food choice experiment to investigate potential egg-predation of Helix. To assess potential influences of environmental conditions and biotic interactions in the greenhouse experiment, we manipulated soil humidity and earthworm activity.

### Mesocosm experiment

We carried out a three-factorial mesocosm experiment between March and May 2016. Factors included were snail/slug presence (3 levels: only *H. pomatia*, only *A. vulgaris*, both *H. pomatia* and *A. vulgaris* present), watering regime (2 levels: addition of 150 ml tap water every day, addition of 450 ml every third day) and earthworm presence (2 levels: addition of *Lumbricus terrestris*, no earthworms). Every combination of factors was replicated 5 times, resulting in totally 60 mesocosms in a Latin row-column design. *A. vulgaris* individuals were collected in gardens in Vienna in March 2016, *H. pomatia* individuals were obtained from a commercial breeder in Vienna (Gugumuck–Wiener Schneckenmanufaktur; https://gugumuck.com/). Since *A. vulgaris* usually lives for one year and dies after egg-laying in autumn, the slugs were still adolescent at the time of the experiment. *H. pomatia*, lives for several years and speciemens used in our experiment were adult.

The mesocosms (35.5 l volume, diameter 35.5 cm, depth 36 cm) were located in a greenhouse of the University of Natural Resources and Life Sciences Vienna in Groß Enzersdorf and were equally filled with substrate which consisted of a mixture of peat-free commercial plant substrate “Guter Grund” and topsoil from an arable field of the university’s research farm. Mesocosms were planted with 3 lettuce seedlings (*Lactuca sativa var. capitate;* mean weight 17.45 g) each in a consistent pattern on 23^rd^ of March; seedlings were obtained from a local gardening shop (Gartenbauer Auer, Vienna, Austria). For the mesocosms with earthworm presence as a factor, 2 individuals of adult *L. terrestris* purchased in a local fisherman shop (Anglertreff Thomas Lux, Vienna, Austria) were added after recording their initial weight (4.82 ± 1.16 g per mesocosm, mean ± SD). During a 27-day adaptation phase for lettuce seedlings and earthworms, all mesocosms were watered daily with 150 ml tap water each. After this establishment period, a stable isotope solution (^15^N) was applied once onto lettuce leaves using a fine brush following the method by Putz et al. (2011). Marking lettuce leaves with stable isotopes made it possible to assess herbivory on a species level, even in those mesocosms, that contained both *H. pomatia* and *A. vulgaris*. The isotopes are taken up by the gastropods together with the lettuce and accumulate in their bodies. The isotope concentrations in the gastropod bodies then gives an indication of how much lettuce was consumed by each specimen during the experiment. We used a 97 atom% ^13^C, 2 atom% ^15^N urea solution, which was produced by dissolving 100 mg 99 atom% ^13^C urea and 2 mg 98 atom% ^15^N urea (Sigma Aldrich, Vienna, Austria) in 50 ml distilled water. Furthermore, we added 12.5 µl wetting agent (Neo-Wett, Kwizda, Vienna, Austria) to ensure good contact of the labelling solution with the leaf surface. The urea solution was carefully applied with the fine brush on both sides of each leaf to avoid contamination of the soil within the mesocosms. Afterwards the different watering regimes were maintained (150 ml resp. 450 ml of water for the respective mesocosms). The water was applied using a long neck to avoid water spilling over the leaves and washing away the urea solution.

Subsequently, *A. vulgaris* and *H. pomatia* individuals were added to the mesocosms 28 days after planting (20^th^ of April); *A. vulgaris*-only mesocosms contained 5 individuals (1.03 g ± 0.16), *H. pomatia*-only mesocosms contained 4 individuals (20.06 g ± 1.86), and mesocosms with both species contained 3 *A. vulgaris* (0.85 g ± 0.17) and 2 *H. pomatia* individuals (19.05 g ± 1.92) each. Before introduction and after a 24-hour starvation period, all slugs and snails were weighed individually.

The experiment was conducted for 14 days. During this period, mesocosms were surveyed every day. In mesocosms where lettuce plants were consumed completely, single, similar-sized lettuce leaves were provided as food in order to prevent starvation of gastropods. These leaves where labelled with urea solution as well. A table with detailed information on the added leaves can be found in the Supplemental Material (Table S1). Soil temperature, soil humidity and soil electric conductivity were recorded during the experiment using a handheld TDR-system (TRIME -PICO 64/32, HD2-hand held device; IMKO Micromodultechnik, Ettlingen, Deutschland) on four dates (April 23^rd^, 24^th^, 25^th^; May 2^nd^ 2016). For the analyses, TDR measurements per mesocosm were averaged.

After the experiment was terminated (May 3^rd^, 2016), we estimated lettuce herbivory (%) visually, and remaining lettuce plants were harvested and dried at 60 °C for 24 h and weighed. Earthworm individuals were collected, counted and weighed. *Arion* and *Helix* individuals were collected, counted, weighed and subsequently frozen at −18 °C. All samples (i.e. all slug and snail individuals and a random selection of salad leaves from four mesocosms) were freeze-dried for 72 h and analysed for their stable isotope signature at the Federal Institute of Education and Research Francisco Josephinum, Wieselburg, Austria. The isotopic composition of C (δ^13^C) and N (δ^15^N) was measured using a Delta V Isotope Ratio Mass Spectrometer, connected via a ConFlo IV interface with an elemental analyser (Thermo Fisher Scientific, Waltham, MA, USA). Between 0.95 and 1 mg of the sample material was introduced into tin capsules for C- and N -isotope analysis. Stable isotope values were expressed applying the conventional δ-notation in parts per thousand (‰), relative to C and N reference materials, as the Vienna Pee Dee Belemnite (VPDB) and on atmospheric N_2_ (AIR), respectively, as follows:

δX‰=((Rsample−Rstandard)/Rstandard)∗1,000

where X is ^13^C or ^15^N and R is the ratio of ^13^C/^12^C and ^15^N/^14^N in each case. Replicate measurements of certified as well as internal laboratory standards show that the measurement errors for both carbon and nitrogen isotope analyses were < ±0.2‰. Sample measurements were carried out at least in duplicates.

To account for the weight difference between *A. vulgaris* and *H. pomatia* individuals upon introduction, we calculated the percentage of herbivory per mesocosm by the total initial weight of all gastropods in each mesocosm and used this as a response variable.

### Food choice experiment

We set up a food choice experiment to test whether *Helix* consumes *Arion* eggs. A total of 200 eggs were collected in private gardens in the Austrian provinces of Vorarlberg and Lower Austria. However, eggs from Lower Austria were only used in three repetitions; the other 37 repetitions with eggs as food choice contained eggs collected from Vorarlberg. *Helix* individuals were again obtained by the commercial breeder Gugumuck–Wiener Schneckenmanufaktur. In the experiment we either offered lettuce leaves (2 g of *Lactuca sativa var. capitate)*, 5 *Arion* eggs or both leaves and eggs to one *Helix* individual (mean weight 22.14 g ± 5.28) and repeated each setting 20 times, resulting in 60 samples in total.

For acclimation and starvation, we kept *H. pomatia* individuals for 24 h on wet paper towels in a transparent plastic container (30 × 20 × 10 cm; L × W × H). After 24 h of starving the snails were weighed, the paper towels were removed, and we offered the different food choices. No soil was added to the container. The containers were kept in a climate chamber at 22 °C with a 12 h day/night rhythm. Additionally, 30 ml of water were added to each box. After 24 h, snails and remaining food were weighed and counted.

### Statistical analyses

For the statistical analyses we used R (Version 3.5.1) and R Studio (Version 1.1.456) (R Core Team, 2018). We calculated a correlation matrix and the variance inflation factor to ensure explanatory variables were not highly correlated. We used Shapiro Wilk tests and additionally quantile-comparison-plots to check for normal distribution of data and residuals respectively. Homogeneity of variances was checked using the Fligner–Killeen test.

We ran a linear model including soil humidity, soil electric conductivity, soil temperature, water regime and earthworm presence as explanatory variables, added an interaction with the factor species for each variable and subsequently ran a Tukey post-hoc test using the glht-function of the R-package “multcomp” (Version 1.4-8). For analysing differences in intraspecific gastropod weight, we used a Kruskal Wallis test.

For weight difference between *Arion* and *Helix*-individuals, we ran ANOVAs after checking for normal distributions of weight differences and homogeneity of variances. To account for the different sizes of the two species, we estimated the percentage of leaves eaten in each mesocosm and divided this percentage by the initial gastropod body mass per mesocosm. Since this percentage of herbivory per gastropod body mass was not normally distributed, we conducted a generalized linear model with a Poisson distribution since the calculated percentages were very low and checked for a constant variance of the standardised residuals.

For the results of the stable isotope analysis, we performed an ANOVA with a Tukey post-hoc test for ^15^N and 13C to determine differences between the different levels of interaction. For ^13^C we had to log-transform the data first. Although ^13^C was still not normally distributed, the Fligner–Killeen test showed homogeneity of variances; therefore, we decided to proceed with an ANOVA. We used the same explanatory variables as in the previous analysis. To account for potential species-specific differences in the uptake of ^13^C and ^15^N, respectively, we used the mean ^13^C/^12^C and ^15^N/^14^N ratios of the salad-samples as a baseline. In a next step we calculated the difference between the isotope ratios of our salad samples and the ratios of the gastropods. In a final step we divided the ^15^N differences through the ^13^C differences for the gastropod samples for each mesocosm. This procedure allowed us to get one value for each gastropod sample that represents the isotope uptake for both ^13^C and ^15^N combined, which will be called “overall isotope uptake”, which is similar to what Aberle et al. (2005) did in their investigation.

## Results

### Mesocosm experiment

*Helix* individuals weighed more than *Arion* individuals upon introduction regardless of the different treatment levels (mean weight per individual ± SD per mesocosm across all treatments: *Arion* 0.938 g ± 0.188; *Helix* 19.556 g ± 1.934). As a result, *Helix* performed significantly more overall herbivory compared to *Arion* (*p* = 0.001), but not compared to mesocosms where both species were present (*p* = 0.573). No effect of watering regime could be detected (*p* = 0.665).

**Figure 1:**
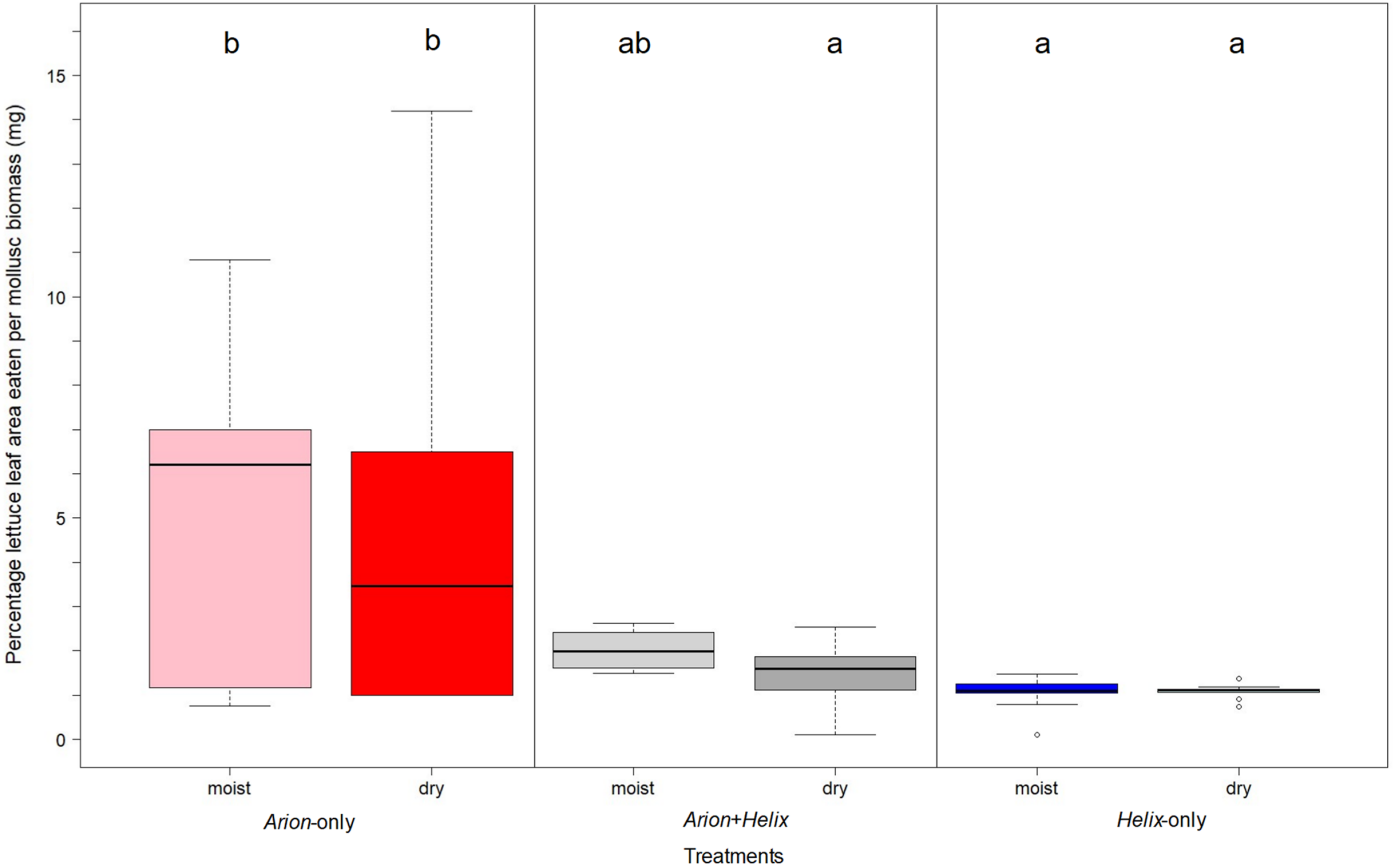
Herbivory on lettuce leaves per gastropod-biomass in *Arion*-only (pink and red), *Arion* and *Helix* (lightgrey and darkgrey), *Helix*-only (blue and lightblue) mesocosms for daily watering and watering every 3 days. Moist = mesocosms with daily watering, dry = mesocosms with watering every three days. Treatments sharing the same letter are not significantly different.

Considering herbivory per bodymass, *Arion*-only significantly surpassed *Helix*-only (*p* < 0.001) and the *Arion* and *Helix* mixed mesocosms (*p* < 0.01) in the performance of lettuce herbivory, irrespective of the watering regime applied (Fig. 1). However, the percentage of herbivory per body mass was significantly different in mesocosms containing both *Arion* and *Helix* compared to *Arion*-only mesocosms when watered every three days (*p* = 0.022).

When analysing environmental factors in the mesocosm experiment (earthworm presence, soil temperature, soil electrical conductivity and soil humidity) in interaction with the respective species we could not detect significant differences. Therefore, the following graphs show results without explicitly presenting these environmental factors as well. However, soil humidity was significantly influenced by watering regime (*p* = 0.022), resulting in a higher soil humidity in mesocosms with daily watering.

*Helix* specimens in average lost weight during the course of the experiment while *Arion* specimens gained weight (mean weight difference per individual ± SD: *Helix* -0.44 g ± 0.95, *Arion* +0.65 g ± 0.33). Weight gain in *Arion* was higher (*p* = 0.001) and weight loss in *Helix* (*p* = 0.010) was lower under daily irrigation than under irrigation every three days.

Overall, no interaction between *Arion* and *Helix* in regard to weight difference could be detected. Individuals of both species showed no significant difference in intraspecific weight at the end of the experiment no matter if they were combined with the other species or not (*Arion*: *p* = 0.82; *Helix*: *p* = 0.91). However, initial weight in both species was significantly lower (*Arion*: *p* = 0.002; *Helix*: *p* = 0.04) at the beginning of the experiment between mesocosms with both gastropods present and mesocosms with either *Arion* or *Helix* present.

The mean isotope ratios for ^15^N and ^13^C for *Arion*, *Helix* and the salad samples are presented in Table 1.

**Table 1:** Stable isotope ratios for ^15^N and ^13^C for *Arion* slugs, *Helix* snails and *Lactuca* lettuce samples across treatments. Means ± SD.

Sample	^15^N ± SD (‰)	^13^C ± SD (‰)
*Arion*	6.346994 ± 1.4201437	−26.49365 ± 1.263899
*Helix*	6.816275 ± 0.9148187	−24.60540 ± 1.346667
*Lactuca*	14.7735 ± 1.847070	−31.123525 ± 0.433338

**Figure 2:**
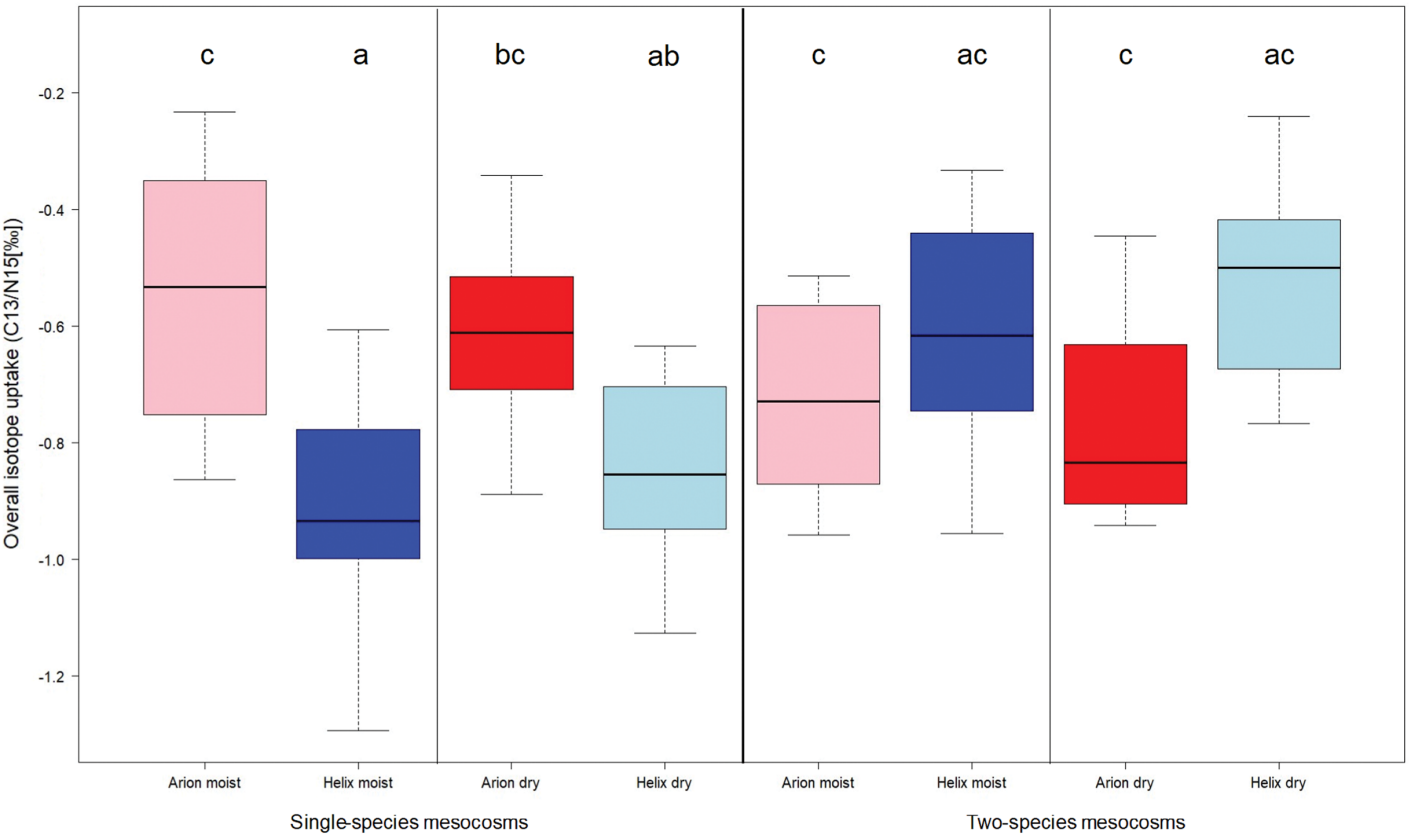
Overall isotope uptake in *Arion* and *Helix* gastropods in single-species mesocosms (left) or two-species mesocosms (right) either with daily watering (moist) or watering every three days (dry). Treatments sharing the same letter are not significantly different.

The analysis of the ^15^N stable isotope concentrations revealed a significant influence of watering regime (*p* = 0.04), the percentage of leaves eaten in a mesocosm (*p* = 0.010) and of an interaction between soil temperature and the presence of both gastropod species in a mesocosm (*p* = 0.010; see Table S2). In contrast, the analyses of the ^13^C-ratios showed significant influences of species (*p* < 0.01), weight difference of the molluscs between the beginning and the end of the experiment (*p* < 0.01) and soil electric conductivity (*p* = 0.011; see Table S3). After calculating the overall isotope uptake to get a more complete picture about isotope uptake by gastropods in general, we conducted an ANOVA and found a significant difference in gastropod species (*p* < 0.01), but no influence of co-occurrence or watering regime (Fig. 2).

Additionally, we found a significant influence of mollusc weight between the beginning and the end of the experiment and the percentage of leaves eaten in a mesocosm (Table 2).

**Table 2:** ANOVA results on overall isotope uptake within molluscs.

Variation	Df	Sum Sq	Mean Sq	F value	*p* (>F)	
Co-occurrence	1	0.1125	0.1125	2.75	0.102165	
Watering regime	1	0.0316	0.0316	0.772	0.383028	
Earthworm presence	1	0.0156	0.0156	0.382	0.538628	
Soil electric conductivity	1	0.1408	0.1408	3.441	0.068188	
Soil humidity	1	0.0055	0.0055	0.134	0.715465	
Soil temperature	1	0.073	0.073	1.785	0.186328	
Weight difference	1	0.5867	0.5867	14.341	0.000339	***
Percentage of herbivory	1	0.1642	0.1642	4.013	0.049376	*
Co-occurrence:watering regime	1	0.0006	0.0006	0.016	0.900862	
Co-occurrence:earthworm presence	1	0.111	0.111	2.713	0.104443	
Co-occurrence:soil electric conductivity	1	0.0013	0.0013	0.032	0.859454	
Co-occurrence:soil humidity	1	0.0008	0.0008	0.02	0.887958	
Co-occurrence:soil temperature	1	0.0205	0.0205	0.502	0.481279	
Co-occurrence:weight difference	1	0.0312	0.0312	0.763	0.385611	
Co-occurrence:percentage of herbivory	1	0.0402	0.0402	0.982	0.325466	
Residuals	64	2.6182	0.0409			

**Note:**

Significant factors are indicated by * for *p* < 0.05, ** for *p* < 0.01 and *** for *p* < 0.001.

### Food choice experiment

We found very little egg predation of *Arion* eggs by *Helix*. *Helix* had been offered five eggs each in 60 repetitions, but only a total of three eggs were eaten in three different containers. In all three cases eggs had only been eaten when lettuce was offered to *Helix* as well. The lettuce was eaten completely in all 40 repetitions containing lettuce. No significant influence of food choice options on *Helix* weight differences between the beginning and the end of the experiment could be detected. We conducted no further statistical analysis due to insufficient data.

## Discussion

Anecdotes circulating among gardeners suggest a negative effect of *Helix* snails on *Arion* slugs. However, the results of our investigation could not clearly confirm such an influence of *H. pomatia* on *A. vulgaris* (or *vice versa*) in terms of weight gains/losses, herbivory or egg predation. While several studies focused on direct or indirect negative influences of invasive gastropod species on native gastropod fauna (Kappes, 2006; Rabitsch, 2006; Roth, Hatteland & Solhøy, 2012; Slotsbo et al., 2012; Allgaier, 2015; Hatteland et al., 2015; Zemanova, Knop & Heckel, 2017), so far, no study, to the best of our knowledge, experimentally tested potential interactions between the Roman snail *H. pomatia* and the invasive Spanish slug (*A. vulgaris*) in combination with other biotic and abiotic factors.

In our experiment, *Arion* individuals gained weight and *Helix* individuals lost weight, which can be explained by the fact that the slugs were still adolescent and therefore growing, whereas the snails were adult and lost more weight due to stress in the experimental setting. This is also reflected by the significantly higher herbivory per body mass of *Arion* compared to *Helix*. Overall, *A. vulgaris* had an almost 5 times higher herbivory per body mass than *Helix*.

However, if we take a closer look at the herbivory per gastropod-body mass in the mesocosms which were watered every three days, we can see in mesocosms with both species present the herbivory per body mass was significantly lower compared to the herbivory per body mass in *Arion*-only mesocosms, but not different to *Helix*-only mesocosms. In mesocosms with daily watering, we could not detect any significant differences between mesocosms containing both gastropod species or single-species mesocosms. This might indicate that *Arion* could have been influenced by the presence of *Helix* when water was applied every three days, since herbivory was shifting towards *Helix*-only mesocosm if compared to the situation when mesocosms were watered daily. An additional explanation for this finding could be that we might have had “overcrowding” in the mesocosms and that *A. vulgaris* was less active in two-species mesocosms when already being stressed due to less frequent watering. Although other studies have also used mesocosms of similar sizes (e.g. Symondson et al., 2006; Wurst & Rillig, 2011; Knop & Reusser, 2012; Roth, Hatteland & Solhøy, 2012; Trouvé et al., 2013; Zaller et al., 2013; Korell et al., 2016), those mesocosms did not contain *H. pomatia* together with *A. vulgaris*. In the field this could lead to evasive behavior at least of younger and much smaller *A. vulgaris* when adult *H. pomatia* is nearby. With the design of our experiment we are not able to determine if *Arion* was indeed influenced by *Helix*, or if both species had a reduced herbivory, since we could only measure herbivory per mesocosm directly. To account for this fact, we additionally used stable isotopes to get a more direct measuring of herbivorous activity by gastropod species.

Stable isotope tracing showed ambiguous results. The analyses of the uptake of the individual isotopes indicate a significant influence of watering regime, the percentage of leaves eaten in a mesocosm and an interaction between the co-occurrence of the gastropod species and soil temperature on ^15^N ratios; ^13^C ratios were significantly influenced by the gastropod species and the weight difference of the gastropods between the beginning and the end of the experiment. The overall isotope uptake was significantly influenced by species and weight difference in gastropods between the beginning and the end of the experiment and the percentage of leaves eaten in a mesocosm. Overall isotope uptake, which included both the ^15^N and ^13^C ratios, indicates that there was no influence of *Helix* on *Arion* or *vice versa*, since we could only detect significant results in weight difference of gastropods, percentage of salad leaves eaten in a mesocosm and species. There was a slight trend of reduced overall isotope uptake in *A. vulgaris* in single-species mesocosms compared to two-species mesocosms, which could develop into a significant difference if the experiment would have lasted longer.

There can be several explanations for these ambiguous results. Since *Helix*-individuals were on average 20 times heavier than *Arion*-individuals (mainly due to the shells), the isotope tracing could not have been sensitive enough to detect the differences in *Helix* over the course of the experiment (Vander Zanden et al., 2015). However, we did find an influence in ^15^N ratios regarding watering regime, percentage of leaves eaten in a mesocosm and to an interaction between the co-occurrence of the gastropod species and soil temperature, suggesting that isotope tracing was indeed working. Additionally, the results of the ^13^C ratios, which were influenced by the weight difference in molluscs and the percentage of salad leaves eaten in a mesocosm, indicate that isotopes were indeed taken up by the gastropods in sufficient amounts.

Another explanation for the ambiguous results could be different isotope turnovers for C and N in different tissues in both species (Crowley et al., 2010). Since *Helix* and *Arion* differ in tissue composition (e.g. shell), the turnover of isotopes could be equally different. Furthermore, different metabolizing pathways for C and N could have additional effects on our results. When we only consider the ^15^N ratios, only *A. vulgaris*, but not *H. pomatia* seems to have benefitted from a more frequent watering regime. Although *Arion* has been shown to cope well with droughts (Slotsbo et al., 2011), reduction in slug activity at dry conditions are reported (South, 1992; Grimm & Kaiser, 2000; Sternberg, 2000; Slotsbo et al., 2011). Snails like *H. pomatia* on the other hand, are less affected by drought periods than slugs, because their shell protects them at least partly against water loss (Pollard, 1975; Thompson et al., 2006; Nicolai et al., 2011; Nicolai & Ansart, 2017). Gastropod species in general are highly influenced by humidity (Crawford-Sidebotham, 1972; Young, Port & Green, 1993). Previous studies have shown that the activity (Pollard, 1975; Hommay, Lorvelec & Jacky, 1998; Grimm & Kaiser, 2000; Sturm, 2007; Kozłowski et al., 2011) and distribution (Barnes & Weil, 1944; Willis et al., 2006; Capinha et al., 2014; Dörler et al., 2018) of gastropods is significantly correlated with humidity, among other environmental factors, which would explain these results.

Since we applied the stable isotope solution directly on the leaves, one possible explanation could be that *Arions* were less active due to drought stress, hiding in shaded and moist areas of the mesocosm and were thus eating less lettuce marked with stable isotopes. *Helix* on the other hand did not reduce its activity under dry conditions and continued to consume labelled lettuce leaves. However, we did not observe any significant decrease in herbivory when watered every 3 days.

The significant interaction between soil temperature and the co-occurrence of both gastropod species in a mesocosms can be explained by the fact the watering regime had an effect on soil temperature, and that the influence of watering regime on *Arion* was more pronounced in two-species mesocosms than in single-species mesocosms.

Former studies showed a negative influence of earthworm activity on slug herbivory (Trouvé et al., 2013; Zaller et al., 2013). In contrast to these studies we found no effect of earthworm activity on slug or snail herbivory, which is also in line with other investigations that considered potential effects of earthworm activity on herbivory (Dörler et al., 2018; Dörler, Scheucher & Zaller, 2019). However, previous investigations showing no effects of earthworms were conducted in the field and did not assess slug herbivory per se, but rather slug occurrences in relation to earthworm activity in gardens (Dörler et al., 2018). Furthermore, we used lettuce plants in our investigation, whereas former studies used a variety of native grassland species which could have reacted more sensitive on earthworm activity (Trouvé et al., 2013; Zaller et al., 2013).

Furthermore, we observed only minor egg-predation of adult *Helix* on eggs of *Arion*. Our finding that only three eggs out of totally 200 eggs offered were eaten only in settings where also lettuce was offered suggests no added nutritional benefit of eggs over lettuce. The three eggs were probably randomly eaten when eggs were sticking to the lettuce leaves. If we look at other studies which investigated egg-cannibalism of *Helix* (Baur, 1988, 1990a), we see that in those studies freshly-hatched *Helix* were investigated, whereas we used adult specimens of *H. pomatia* in our experiment. If egg-cannibalism or -predation is confined to the early live stages of *Helix*, our experimental setting cannot detect this influence. In the field Arion eggs are placed under vegetation or stones (Slotsbo et al., 2011), which in many cases can hardly be accessed by big snails with shells. Therefore, egg predation by young *H. pomatia* would probably have a more significant influence than by adult individuals, if it happens at all.

Based on these first insights into interactions between *H. pomatia* and *A. vulgaris* we recommend further, longer-running experiments using larger units of field plots. A special focus could be laid on avoidance behavior and environmental factors, as we could only account for them in a very limited way in our experimental design. Furthermore, future food-choice-experiments could also study the behaviour of juvenile *Helix* or potential egg predation of *Helix* eggs by *Arion* species.

## Conclusions

This was a first attempt to understand ecological interactions between the snail *H. pomatia* and the invasive slug *A. vulgaris*. Although not significant, our results indicated some interference of the two gastropod species at least under drier conditions, precluding us to dismiss the anecdotal evidence that adult *H. pomatia* is negatively affecting the invasive Spanish slug *A. vulgaris*. However, we found no convincing evidence that this interference might also include the predation of *Helix* on *Arion* eggs or the interaction with earthworms. Sustaining high populations of *H. pomatia* seem to be able to pose some stress to invasive *A. vulgaris* under certain conditions, however, further experiments in a more realistic setting would be necessary to elucidate underlying functional links.

## Supplemental Information

10.7717/peerj.11309/supp-1 Supplemental Information 1 Measurements of *A. vulgaris* and *H. pomatia* during the mesocosm experiment.

10.7717/peerj.11309/supp-2 Supplemental Information 2 Supplemental tables on additional food provided and ANOVA results of ^13^C and ^15^N-isotope ratios.

10.7717/peerj.11309/supp-3 Supplemental Information 3 Raw data and measurement for egg predation experiment. All factors and measurements that are the basis of the statistical analyses.

## Additional Information and Declarations

### Data Availability

The following information was supplied regarding data availability: The raw measurements are available in the Supplemental Files.
