# Supplementary material for: Experimental indications of gardeners’ anecdotes that snails interfere with invasive slugs
Source: PeerJ. 2021 May 11;9:e11309. doi: 10.7717/peerj.11309 (PMC8121057; doi:10.7717/peerj.11309)
Supplement: Supplemental Information 2 [file peerj-09-11309-s002.docx]

**Supplemental Table S1:**

**List of lettuce leaves added to mesocosms when planted salad was completely eaten**

| **Date of feeding** | **Mesocosm number** | **Number of lettuce leaves** | **Weight (g)** |
| --- | --- | --- | --- |
| 24.04.2016 | 5 | 1 | 3.36 |
| 24.04.2016 | 24 | 1 | 3.44 |
| 24.04.2016 | 27 | 1 | 3.22 |
| 24.04.2016 | 38 | 1 | 3.1 |
| 24.04.2016 | 34 | 1 | 3.45 |
| 24.04.2016 | 52 | 1 | 3.33 |
| 25.04.2016 | 5 | 1 | 2.25 |
| 25.04.2016 | 24 | 1 | 2.54 |
| 25.04.2016 | 27 | 1 | 2 |
| 25.04.2016 | 28 | 1 | 2.21 |
| 25.04.2016 | 40 | 1 | 2.49 |
| 25.04.2016 | 38 | 1 | 2.53 |
| 25.04.2016 | 34 | 1 | 2.44 |
| 25.04.2016 | 52 | 1 | 2.76 |
| 25.04.2016 | 53 | 1 | 2.51 |
| 26.04.2016 | 5 | 1 | 4 |
| 26.04.2016 | 24 | 1 | 3.85 |
| 26.04.2016 | 27 | 1 | 4.07 |
| 26.04.2016 | 28 | 1 | 4.37 |
| 26.04.2016 | 40 | 1 | 4.1 |
| 26.04.2016 | 38 | 1 | 4.3 |
| 26.04.2016 | 34 | 1 | 3.75 |
| 26.04.2016 | 52 | 1 | 3.38 |
| 26.04.2016 | 53 | 1 | 3.91 |
| 27.04.2016 | 5 | 1 | 4.18 |
| 27.04.2016 | 23 | 1 | 4.97 |
| 27.04.2016 | 24 | 1 | 4.74 |
| 27.04.2016 | 27 | 1 | 4.63 |
| 27.04.2016 | 28 | 1 | 4.96 |
| 27.04.2016 | 40 | 1 | 4.91 |
| 27.04.2016 | 38 | 1 | 4.89 |
| 27.04.2016 | 34 | 1 | 4.92 |
| 27.04.2016 | 51 | 1 | 4.5 |
| 27.04.2016 | 52 | 1 | 4.54 |
| 27.04.2016 | 53 | 1 | 4.33 |
| 28.04.2016 | 5 | 1 | 4.06 |
| 28.04.2016 | 23 | 1 | 3.77 |
| 28.04.2016 | 24 | 1 | 3.96 |
| 28.04.2016 | 27 | 1 | 3.99 |
| 28.04.2016 | 28 | 1 | 3.87 |
| 28.04.2016 | 34 | 1 | 3.97 |
| 28.04.2016 | 38 | 1 | 3.96 |
| 28.04.2016 | 40 | 1 | 3.57 |
| 28.04.2016 | 51 | 1 | 3.89 |
| 28.04.2016 | 52 | 1 | 3.91 |
| 28.04.2016 | 53 | 1 | 3.83 |
| 29.04.2016 | 5 | 1 | 3.66 |
| 29.04.2016 | 28 | 1 | 3.38 |
| 29.04.2016 | 27 | 1 | 4 |
| 29.04.2016 | 24 | 1 | 3.74 |
| 29.04.2016 | 23 | 1 | 3.54 |
| 29.04.2016 | 34 | 1 | 3.85 |
| 29.04.2016 | 38 | 1 | 3.59 |
| 29.04.2016 | 40 | 1 | 3.81 |
| 29.04.2016 | 51 | 1 | 3.4 |
| 29.04.2016 | 52 | 1 | 3.92 |
| 29.04.2016 | 53 | 1 | 3.59 |
| 30.04.2016 | 5 | 1 | 5.2 |
| 30.04.2016 | 24 | 1 | 5.1 |
| 30.04.2016 | 27 | 1 | 5.1 |
| 30.04.2016 | 28 | 1 | 5.2 |
| 30.04.2016 | 34 | 1 | 5 |
| 30.04.2016 | 38 | 1 | 5.3 |
| 30.04.2016 | 40 | 1 | 5.1 |
| 30.04.2016 | 51 | 1 | 5 |
| 30.04.2016 | 52 | 1 | 5.1 |
| 30.04.2016 | 53 | 1 | 5.3 |
| 30.04.2016 | 23 | 1 | 5 |
| 01.05.2016 | 5 | 1 | 4.4 |
| 01.05.2016 | 23 | 1 | 4.2 |
| 01.05.2016 | 24 | 1 | 4.5 |
| 01.05.2016 | 27 | 1 | 4.3 |
| 01.05.2016 | 28 | 1 | 4.2 |
| 01.05.2016 | 34 | 1 | 4.4 |
| 01.05.2016 | 38 | 1 | 4.4 |
| 01.05.2016 | 40 | 1 | 4.4 |
| 01.05.2016 | 51 | 1 | 4.1 |
| 01.05.2016 | 52 | 1 | 4.2 |
| 01.05.2016 | 53 | 1 | 4.3 |

**Supplemental Table S2:**

**ANOVA results on ^15^N isotope concentrations within molluscs.** Significant factors are indicated by * for p < 0.05 and ** for p < 0.01.

| **Variation** | **Df** | **Sum Sq** | **Mean Sq** | **F value** | **p (>F)** |  |
| --- | --- | --- | --- | --- | --- | --- |
| *Arion* and *Helix* co-occurrence | 1 | 0.38 | 0.381 | 0.3 | 0.58584 |  |
| Watering regime | 1 | 5.46 | 5.463 | 4.296 | 0.04224 | * |
| Earthworm presence (EW) | 1 | 0.25 | 0.253 | 0.199 | 0.65722 |  |
| Soil electric conductivity (SEC) | 1 | 0.8 | 0.799 | 0.628 | 0.43105 |  |
| Soil humidity | 1 | 4.2 | 4.198 | 3.301 | 0.07392 |  |
| Soil temperature | 1 | 0.02 | 0.021 | 0.016 | 0.89821 |  |
| Weight difference (initial-end) | 1 | 0.87 | 0.867 | 0.682 | 0.41205 |  |
| Percentage of herbivory | 1 | 9.03 | 9.029 | 7.1 | 0.00975 | ** |
| Co-occurrence:watering regime | 1 | 0.25 | 0.248 | 0.195 | 0.66031 |  |
| Co-occurrence:EW | 1 | 1.74 | 1.744 | 1.372 | 0.24589 |  |
| Co-occurrence:SEC | 1 | 0.04 | 0.039 | 0.031 | 0.86184 |  |
| Co-occurrence:soil humidity | 1 | 0.39 | 0.387 | 0.304 | 0.58314 |  |
| Co-occurrence:soil temperature | 1 | 8.99 | 8.991 | 7.069 | 0.00989 | ** |
| Co-occurrence:weight difference | 1 | 1.71 | 1.706 | 1.341 | 0.25113 |  |
| Co-occurrence:herbivory (%) | 1 | 0.18 | 0.178 | 0.14 | 0.70981 |  |
| Residuals | 64 | 81.39 | 1.272 |  |  |  |

**Supplemental Table S3:**

**ANOVA results on ^13^C isotope concentrations within molluscs.** Significant factors are indicated by * for p < 0.05.

| **Variation** | **Df** | **Sum Sq** | **Mean Sq** | **F value** | **p (>F)** |  |
| --- | --- | --- | --- | --- | --- | --- |
| *Arion* and *Helix* co-occurrence | 1 | 0.0281 | 0.02815 | 3.275 | 0.07505 |  |
| Watering regime | 1 | 0.001 | 0.00099 | 0.115 | 0.735147 |  |
| Earthworm presence (EW) | 1 | 0.0001 | 0.00006 | 0.007 | 0.933533 |  |
| Soil electric conductivity (SEC) | 1 | 0.0584 | 0.0584 | 6.794 | 0.011365 | * |
| Soil humidity | 1 | 0.0044 | 0.00442 | 0.514 | 0.475961 |  |
| Soil temperature | 1 | 0.0131 | 0.01309 | 1.523 | 0.221645 |  |
| Weight difference (initial-end) | 1 | 0.1451 | 0.1451 | 16.88 | 0.000115 | *** |
| Percentage of herbivory | 1 | 0.0045 | 0.00453 | 0.527 | 0.470563 |  |
| Co-occurrence:watering regime | 1 | 0.0009 | 0.00095 | 0.11 | 0.740911 |  |
| Co-occurrence:EW | 1 | 0.0263 | 0.02634 | 3.064 | 0.084846 |  |
| Co-occurrence:SEC | 1 | 0.000 | 0.000 | 0.000 | 0.992832 |  |
| Co-occurrence:soil humidity | 1 | 0.0042 | 0.00424 | 0.493 | 0.485013 |  |
| Co-occurrence:soil temperature | 1 | 0.0019 | 0.00187 | 0.218 | 0.642151 |  |
| Co-occurrence:weight difference | 1 | 0.001 | 0.00102 | 0.119 | 0.731743 |  |
| Co-occurrence:herbivory (%) | 1 | 0.0053 | 0.00527 | 0.613 | 0.436572 |  |
| Residuals | 64 | 0.5501 | 0.0086 |  |  |  |
